# Absence of Rift Valley Fever Virus in Wild Small Mammals, Madagascar

**DOI:** 10.3201/eid1906.121074

**Published:** 2013-06

**Authors:** Marie-Marie Olive, Nadia Razafindralambo, Tony Andrianaivo Barivelo, Jean-Théophile Rafisandratantsoa, Voahangy Soarimalala, Steven M. Goodman, Pierre E. Rollin, Jean-Michel Heraud, Jean-Marc Reynes

**Affiliations:** Institut Pasteur, Antananarivo, Madagascar (M.-M. Olive, N. Razafindralambo, J.-T. Rafisandratantsoa, J.-M. Heraud);; Association Vahatra, Antananarivo (T. Andrianaivo Barivelo, V. Soarimalala, S.M. Goodman);; Université d’Antananarivo, Antananarivo (T. Andrianaivo Barivelo);; Field Museum of Natural History, Chicago, Illinois, USA (S.M. Goodman);; Centers for Disease Control and Prevention, Atlanta, Georgia, USA (P.E. Rollin);; Institut Pasteur, Lyon, France (J.-M. Reynes)

**Keywords:** Rift Valley fever, wild terrestrial mammals, Madagascar, viruses

**To the Editor:** Rift Valley fever virus (RVFV) is a mosquito-borne zoonotic virus in the family *Bunyaviridae*, genus *Phlebovirus*, which affects mainly domestic ruminants and humans on continental Africa, Madagascar, and the Arabian Peninsula (*1*). RVFV is transmitted between ruminants mainly by bites of mosquitoes of several genera (*1*). Infection can lead to mild symptoms or can cause abortion in pregnant animals and high mortality rates among newborns. Humans are mostly infected by aerosol transmission when handling infected tissues (aborted fetuses or meat), which results in dengue-like illness. Some cases in humans can be in a severe form (hemorrhagic fever and meningoencephalitis), which can be fatal. Outbreaks in southern and eastern Africa are associated with periods of heavy rainfall (*1*). In eastern Africa, RVFV is believed to be maintained during interepizootic periods through vertical transmission in *Aedes* spp. mosquitoes (*1*). It has been suspected that wild mammals, especially rodents, play a role in the maintenance of RVFV during interepizootic periods (*2*). However, evidence of a wild mammal reservoir in the epidemiologic cycle of RVFV has yet to be demonstrated (*2*).

In Madagascar, the first RVFV isolate was obtained from mosquitoes captured in the Périnet Forest (Andasibe, Moramanga District) in 1979, outside an epizootic period (*3*). Two epizootic episodes occurred, during 1990–91 and 2008–09 (*4*). After the most recent episode, domestic ruminants were shown to be involved in RVFV circulation during interepizootic periods (*5*,*6*); together with the potential vertical transmission in *Aedes* spp. mosquitoes in Madagascar, they might play a role in the maintenance of RVFV. However, genetic evidence indicates that RVFV outbreaks in Madagascar are not associated with emergence from enzootic cycles but that they are associated with recurrent virus introductions from mainland east Africa (*7*). Although these mechanisms for RVFV epidemiology on Madagascar are documented, the possibility of a wild mammal reservoir cannot be excluded. We therefore explored the role of wild terrestrial small mammals in the maintenance of RVFV in Madagascar, especially the nonnative, abundant, and ubiquitous black rats (*Rattus rattus*) (*8*), as has been suggested in rural Egypt (*9*,*10*).

For this study, 1,610 blood samples were obtained from different species of wild terrestrial small mammals in Madagascar (Figure). Permits to capture and collect animals were obtained from national authorities. Animals were sampled from October 2008 through March 2010 at a site in the Anjozorobe-Angavo (Anjozorobe District) forest corridor (18°18′′41.9′ S, 48°00′′57.6′ E), where RVFV was first detected in humans and cattle in February 2008 (*4*) and within 100 km from where the first RVFV was isolated in 1979 (*3*). We collected 378 serum samples from 11 native Tenrecidae (Afrosoricida) tenrecs, 114 samples from 6 native Nesomyidae (Rodentia) rodents, and 471 samples from introduced *R. rattus* (Muridae, Rodentia) rats (Technical Appendix). In addition, during 2008, we obtained serum samples from 647 *R*. *rattus* or *R*. *norvegicus* rats living near humans in areas where RVFV was reportedly circulating during 2008 and 2009: the districts of Ankazobe, Antsiranana, Betafo, Ihosy, Marovoay, and Moramanga (*4*,*5*) (Figure).

**Figure:**
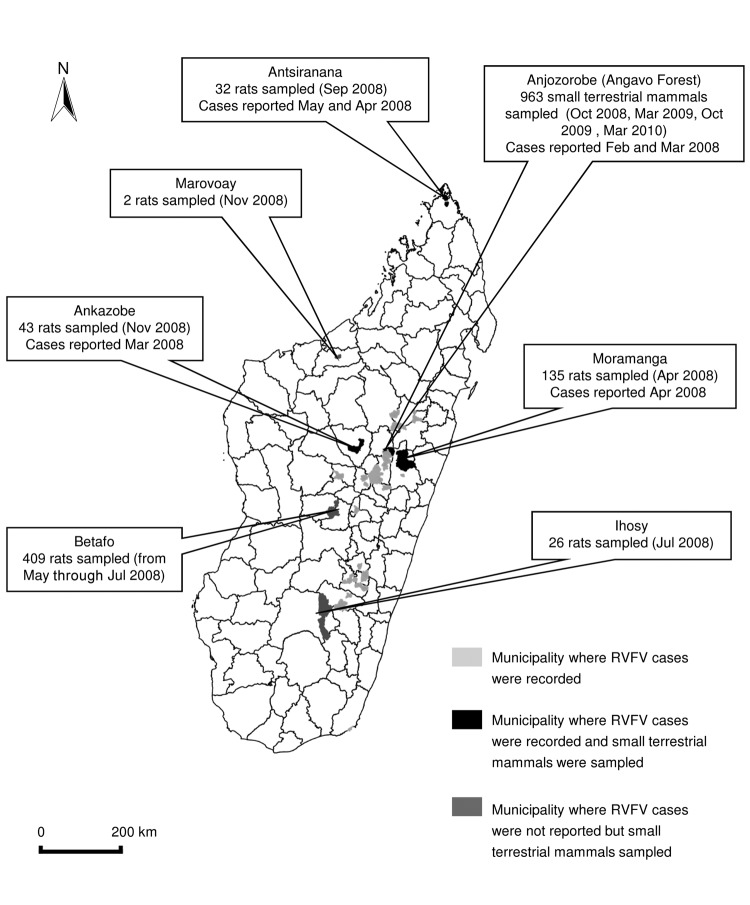
Collection sites of wild terrestrial small mammals on Madagascar and the number of mammals tested for Rift Valley fever virus (RVFV). At certain localities, the genus and species of sampled rats were *Rattus rattus* or *R. norvegicus*.

Serum samples were tested for IgG against RVFV by ELISA, as described (*4*), by using peroxidase-labeled recombinant protein A/G (Pierce, Rockford, IL, USA) or anti-mouse or rat IgG (H+L) according to the ability to recognize the immunoglobulin of species endemic to Madagascar (data not shown). The results were negative for all samples tested. Liver and spleen samples from 947 animals caught in the Anjozorobe-Angavo forest corridor were also tested. Approximately 50–100 mg of liver and spleen from each individual was mixed and homogenized at a dilution of 1:10 in culture medium containing 40% fetal bovine serum. After centrifugation, supernatants were collected and pooled by species (maximum 5 individuals/pool). RNA was extracted from pooled supernatants by using TRIzol LS reagent (Invitrogen, Carlsbad, CA, USA) according to the manufacturers’ instructions. Detection of RVFV RNA was attempted by using real-time reverse transcription PCR (*4*). The results were negative for the 220 monospecific pools tested.

Serologic and virologic results from rodent and tenrec samples collected during and after the epizootic 2008–2009 periods were negative for RVFV; 72.8% had been collected in municipalities where RVFV cases were reported. This finding does not indicate a role of native Rodentia and Afrosoricida mammals in the epidemiology of RVFV in Madagascar, nor does it indicate evidence of infection of *Rattus* spp. rats, as suggested in Egypt (*9*,*10*). The absence of infection in *Rattus* spp. rats during a period of intense RVFV circulation does not support its potential role during the outbreak and, a fortiori, in the maintenance of RVFV during interepizootic periods. Among wild terrestrial mammals in Madagascar, animals of the orders Carnivora and Primata are not considered as candidates for the maintenance of RVFV; however, bats (order Chiroptera) and introduced bushpigs (order Artiodactyla , family Suidae, genus *Potamochoerus*) could be candidates, and their role in RVFV maintenance should be investigated (*2*). At present, no evidence is available for the maintenance of RVFV in wild terrestrial small mammals (native and introduced) in Madagascar.

Technical Appendix Taxonomy of small terrestrial animals sampled in Anjozorobe, Madagascar, October 2008 and 2009, March 2009 and 2010
