# Supplementary material for: Absence of Rift Valley Fever Virus in Wild Small Mammals, Madagascar
Source: Emerg Infect Dis. 2013 Jun;19(6):1025–7. doi: 10.3201/eid1906.121074 (PMC3713820; doi:10.3201/eid1906.121074)
Supplement: Technical Appendix — Taxonomy of small terrestrial animals sampled in Anjozorobe, Madagascar, October 2008 and 2009, March 2009 and 2010 [file 12-1074-Techapp-s1.pdf]

Article DOI: http://dx.doi.org/10.3201/eid1906.121074

Absence of Rift Valley Fever Virus in Wild Mammals, Madagascar

Technical Appendix

Technical Appendix Table. Taxonomy of small terrestrial animals sampled in Anjozorobe, Madagascar, October 2008 and 2009, March 2009 and 2010

Order	Family	Species	No.
Afrosoricida	Tenrecidae	*Hemicentetes semispinosus*	25
		Microgale dobsoni	219
		M. fotsifotsy	3
		M. gymnorhyncha	3
		M. longicaudata/majori	8
		M. parvula	2
		M. thomasi	15
		M. soricoides	61
		Oryzorictes hova	38
		Tenrec ecaudatus	2
		Setifer setosus	2
Rodentia	Nesomyidae	*Eliurus majori*	15
		E. minor	17
		E. tanala	15
		E. grandidieri	3
		Gymnuromys roberti	11
		Nesomys rufus	53
	Muridae	*Rattus rattus*	471
